# Complete Genome Sequence of *Leuconostoc citreum* Strain NRRL B-742

**DOI:** 10.1128/genomeA.01179-14

**Published:** 2014-11-26

**Authors:** Delphine Passerini, Marlène Vuillemin, Sandrine Laguerre, Myriam Amari, Valentin Loux, Valérie Gabriel, Hervé Robert, Sandrine Morel, Pierre Monsan, Bruno Gabriel, Catherine Fontagné-Faucher, Magali Remaud-Siméon, Claire Moulis

**Affiliations:** a Université de Toulouse , INSA, UPS, INP, LISBP, Toulouse, France; b INRA, UMR792, Ingénierie des Systèmes Biologiques et des Procédés, Toulouse, France; c CNRS, UMR5504, Toulouse, France; d Université de Toulouse, Laboratoire de Biotechnologies Agroalimentaire et Environnementale, EA 4565, Institut Universitaire de Technologie, Université Paul Sabatier, Auch, France; e INRA, UR1077 Mathématique, Informatique et Génome, Jouy-en-Josas, France

## Abstract

*Leuconostoc citreum* belongs to the group of lactic acid bacteria and plays an important role in fermented foods of plant origin. Here, we report the complete genome of the *Leuconostoc citreum* strain NRRL B-742, isolated in 1954 for its capacity to produce dextran.

## GENOME ANNOUNCEMENT

*Leuconostoc citreum* is a heterofermentative lactic acid bacterium that plays an important role in many food fermentations. This species is also known for its capacity to produce a large panel of exopolysaccharides (1), in particular homopolymers such as fructans and glucans (2). Recently, four genomic sequences of *L. citreum* have been reported: the assembled genome of strain KM20 isolated from kimchi (3) and the draft genomes of strains E16, C10, and C11 isolated from wheat sourdoughs (4).

First classified as *Leuconostoc mesenteroides*, the strain NRRL B-742 (other collection numbers: ATCC 13146, DSM 20188, KCTC 3524) has been reclassified as *L. citreum*, on the basis of REP-PCR and fermentation profiles (5). These results were confirmed by amplification and sequencing of the V1-V4 region of the 16S rDNA, using primers E8_F (5′ AGAGTTTGATCCTGGCTCAG 3′) and E807_R (5′ TGGACTACCAGGGTATCTAATC 3′) (6). The NRRL B-742 strain was isolated in the middle of the 20th century for its capacity to produce two types of dextran (Jeanes et al. 1954). The first one was described to contain 73% of α-(1 → 6) linkages and 16% of α-(1 → 4) linkages. The second one presents a very original comb-like structure, with 50% of α-(1 → 6) linkages in the main chain, and 50% of branchings with α-(1 → 3) linkages (7, 8).

The genomic DNA of NRRL B-742 was obtained by coupling whole-genome shotgun and 8 kb-paired-end libraries sequencing, using Roche 454 GS FLX Titanium (MWG-Eurofins, Germany). The shotgun sequencing generates 156,948 clipped reads totaling 98,207,010 pb (coverage of 56×) and the paired-end library sequencing generates 231,542 clipped reads totaling 41,535,478 pb (coverage of 23×). All reads (shotgun + pair-end libraries) were assembled with Newbler version 2.6, generating 78 contigs (with 46 contigs over than 1 kb in size) distributed in two scaffolds. The 18 intrascaffold gaps represented only about 13 kb of unknown regions.

The NRRL B-742 genome is composed of one circular chromosome of 1,718,100 pb with a G+C content of 38.9%, and one plasmid of 27,566 pb. Genome annotation was performed using the AGMIAL platform (9), followed by manual inspection concerning regions of interest. Automatic annotation predicted 1,734 CDSs, 3 rRNA operons, and 66 tRNA encoding genes; 16 putative pseudogenes were identified. Phage annotation using the Phast database (10) revealed 3 putative incomplete phages of 6.7 kb, 19.0 kb, and 27.8 kb. The insertion sequences were manually checked from the IS (Insertion Sequences) Finder database (11). The NRRL B-742 chromosome carries 3 types of IS: one entire copy of the IS110 family, 11 copies of the IS30 family (one of which is truncated), and 10 copies of the IS3 family (6 of which are truncated).

### Nucleotide sequence accession numbers.

This whole-genome shotgun project has been deposited in EMBL/ENA under the accession number CCNG00000000 for the chromosome sequence and LN610406 for the plasmid sequence.
